# Detailing neuroanatomical development in late childhood and early adolescence using NODDI

**DOI:** 10.1371/journal.pone.0182340

**Published:** 2017-08-17

**Authors:** Alyssa Mah, Bryce Geeraert, Catherine Lebel

**Affiliations:** 1 Biomedical Engineering Program, University of Calgary, Calgary, Alberta, Canada; 2 Alberta Children’s Hospital Research Institute, and Owerko Centre; University of Calgary, Calgary, Alberta, Canada; 3 Department of Radiology, University of Calgary, Calgary, Alberta, Canada; University Medical Center Utrecht, NETHERLANDS

## Abstract

Diffusion tensor imaging (DTI) studies have provided much evidence of white and subcortical gray matter changes during late childhood and early adolescence that suggest increasing myelination, axon density, and/or fiber coherence. Neurite orientation dispersion and density imaging (NODDI) can be used to further characterize development in white and subcortical grey matter regions in the brain by improving specificity of the MRI signal compared to conventional DTI. We used measures from NODDI and DTI to examine white and subcortical gray matter development in a group of 27 healthy participants aged 8–13 years. Neurite density index (NDI) was strongly correlated with age in nearly all regions, and was more strongly associated with age than fractional anisotropy (FA). No significant correlations were observed between orientation dispersion index (ODI) and age. This suggests that white matter and subcortical gray matter changes during late childhood and adolescence are dominated by changes in neurite density (i.e., axon density and myelination), rather than increasing coherence of axons. Within brain regions, FA was correlated with both ODI and NDI while mean diffusivity was only related to neurite density, providing further information about the structural variation across individuals. Data-driven clustering of the NODDI parameters showed that microstructural profiles varied along layers of white matter, but that that much of the white and subcortical gray matter matured in a similar manner. Clustering highlighted isolated brain regions with decreasing NDI values that were not apparent in region-of-interest analysis. Overall, these results help to more specifically understand patterns of white and gray matter development during late childhood and early adolescence.

## Introduction

Late childhood and adolescence are periods of significant behavioural, emotional, and cognitive development. Underlying these changes are multiple processes of brain maturation, which are critical for proper development. Diffusion tensor imaging (DTI) has provided much insight into brain development, detailing development trends including increasing fractional anisotropy (FA) and decreasing radial diffusivity (RD) with age [1–6], changes suggestive of increasing myelination, axonal packing, and/or axon coherence [7–9]. New imaging techniques can offer more specificity as to types of changes occurring during this maturation. For example, neurite orientation dispersion and density imaging (NODDI) is a multi-component model using MR diffusion data that captures neurite (dendrites and axons) morphology, providing parameters including neurite density index (NDI) and orientation density index (ODI), which provide more insight than DTI into the changing cellular architecture [10]. Neurite density increases suggest myelination, axonal growth, or greater axonal density, while ODI describes the dispersion of the neurites, probing coherence and geometry.

The increased specificity of NODDI offers promise for better understanding brain development, yet only a few studies have used NODDI to study brain development [11–13]. One study showed increasing intra-axonal water in the corpus callosum and posterior limb of the internal capsule in the first 3 years of life without accompanying changes in fiber orientation [13]. Another study used NODDI to examine healthy brain maturation across the white matter in 66 subjects aged 7–63 years, and found that NDI followed a logarithmic growth curve, increasing from childhood into adulthood, while ODI followed an exponential curve that was flat across adolescence and had accelerating increases during adulthood [11]. Specifically in children and adolescents, one study showed stronger relationships between age and NDI than between age and fractional anisotropy (FA) within white matter tracts, suggesting that NDI is a better measure of age-related variation across subjects than FA [12]. Though subcortical gray matter undergoes significant maturation during childhood and adolescence, with large increases in FA and decreases in mean diffusivity (MD) [14], its development has not been as widely studied. One study used NODDI to show that maturation in the thalamus of preterm infants was driven by increases in NDI [15], but gray matter changes later in childhood remain unclear.

Maturation trajectories vary regionally across the brain; callosal white matter matures earliest and frontal-temporal white matter connections mature latest, with developmental changes continuing into young adulthood [2, 4, 16]. A previous NODDI study also observed varying growth rates across white matter categories, with limbic and projection fibers showing the highest rates of change in NDI, though specific tracts were not directly compared and gray matter was not included [11]. The other NODDI study on children and adolescents also showed steeper growth curves for NDI in the superior longitudinal fasciculus than the uncinate or cingulum [12]. Data-driven clustering can identify patterns of brain development and provide insight into brain organization in a way that may not be apparent when examining specific structures. For example, clustering methods have been used in imaging studies to identify patterns of cortical development in childhood and adolescence [17], microstructural maturation in infants [18], and genetic influence on brain structure [19]. One previous NODDI study noted different patterns in subcortical, cortical, and core white matter zones [11]; data-driven clustering may provide more insight into these microstructural patterns.

The goal of this study was to use NODDI and DTI to study white matter and subcortical grey matter changes during normal late childhood and early adolescent development. Using NODDI and data-driven clustering, we can better characterize developmental changes during this period including regional variation, ultimately providing a better understanding of normal brain maturation. As brain development trajectories can be a sensitive marker of abnormalities [20], information provided by this research may be useful for future studies to better understand developmental disorders.

## Methods

### Subject demographics

27 healthy participants (12 F/15 M) aged 8–13 years (mean +/- SD: 11.3 +/- 1.9 years) participated in this study. Informed written assent was obtained from each subject, and his/her parent or guardian provided written informed consent. Exclusion criteria were known diagnosed developmental and learning disorders, history of neurosurgery or head trauma, or any contraindications to MRI. This research was approved by the University of Calgary Conjoint Health Research Ethics Board.

### MRI acquisition

**Fig 1:**
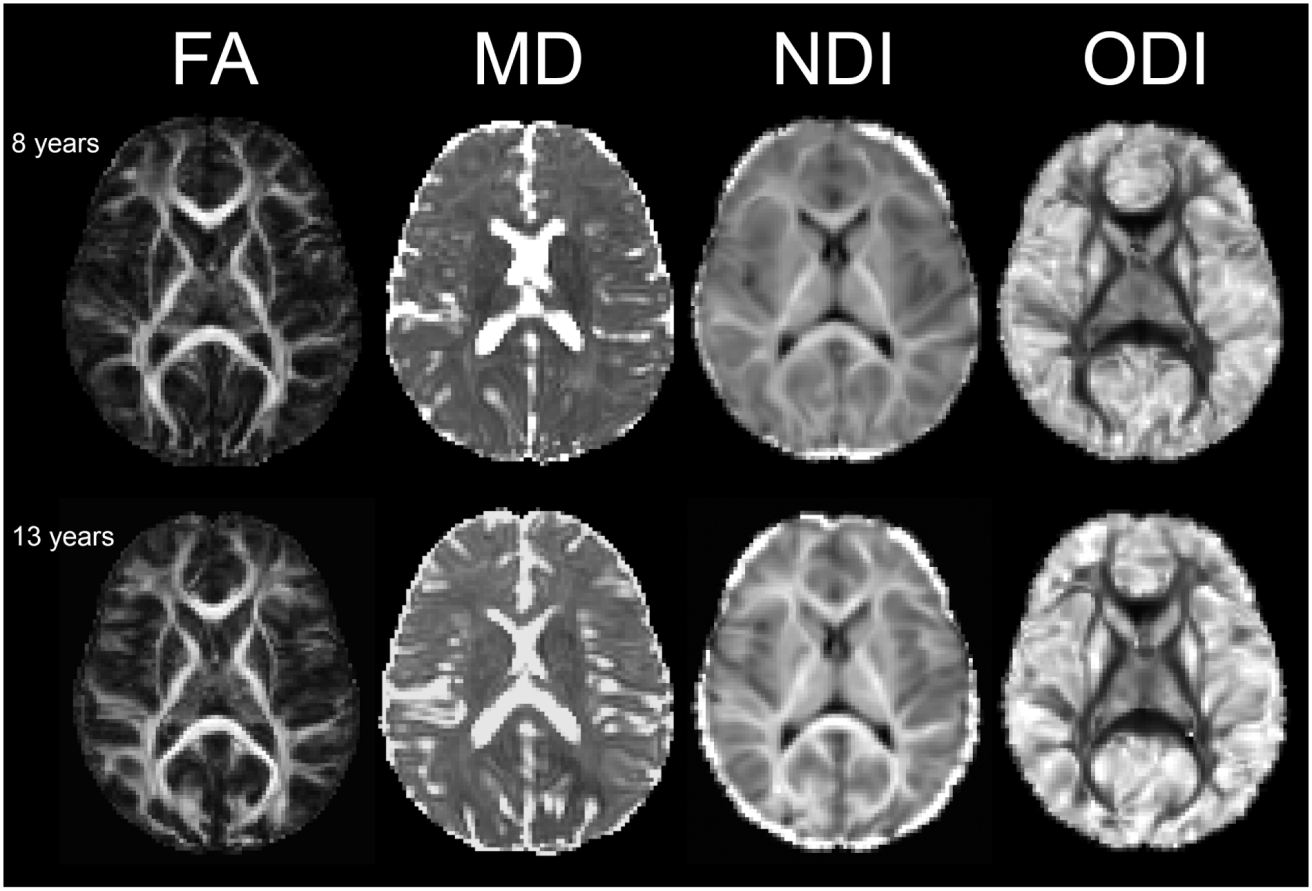
DTI and NODDI measure maps in two healthy participants. Fractional anisotropy (FA), mean diffusivity (MD), neurite density index (NDI), and orientation dispersion index (ODI) are shown in two representative participants. The top row is an 8-year-old male, and the bottom row is a 13-year-old female.

MRI data was collected on a 3T MR system (Discovery 750w; General Electric; Waukesha, WI) using a 32-channel head coil at the Alberta Children’s Hospital. T1-weighted anatomical images were acquired with the following parameters: TI = 600 ms, TR/TE = 8.208/3.156 ms, 0.8 mm^3^ isotropic resolution, scan time = 5:38 min:sec. Diffusion weighted images were acquired using single spin echo EPI with 10 non-diffusion weighted (b = 0 s/mm^2^) images, and two non-zero b-values (900, 2000 s/mm^2^) each with 30 directions; TR/TE = 12 s/88 ms, 2.2 mm^3^ isotropic resolution, total scan time = 14:24 min:sec. All raw and processed data was visually inspected and determined to be of good-to-excellent quality. Fig 1 shows example images from two participants.

### Image analysis

Diffusion-weighted images were eddy current corrected in FSL [21]. Data with b = 0, 900 s/mm^2^ were processed through dtifit to compute the diffusion tensor to obtain FA, MD, RD, and AD maps, and then through bedpostx to fit the probabilistic diffusion model. Diffusion-weighted images from b = 0, 900, and 2000 s/mm^2^ were used to fit to the NODDI model, using the NODDI Matlab Toolbox (http://www.nitrc.org/projects/noddi_toolbox) [10] to provide NDI (fibre volume fraction) and ODI (orientation density index) parameters. Data is available through Figshare: https://figshare.com/s/2f5fe31c7eee30d4da7c.

Advanced Normalization Tools (ANTs) [22] was used to linearly register each subject’s b0 diffusion images to their T1-weighted image, using the default parameters from antsRegistrationSyN.sh. The calculated normalization parameters were then applied to all diffusion parameter maps (FA, NDI, etc.).

T1 data were processed using recon—all in FreeSurfer, and then input to TRACULA [23] along with bedpostx data to automatically segment the following white matter tracts: anterior thalamic radiation (ATR), cingulum, cortical spinal tract (CST), forceps major & minor, inferior longitudinal (ILF), superior longitudinal (SLF), and uncinate fasciculi. These regions were segmented in diffusion space and then transformed to T1-weighted space according to the previously calculated normalization parameters. Subcortical gray matter segmentations of the thalamus, caudate, putamen, pallidum, hippocampus, and amygdala were obtained from FreeSurfer in T1 space. Average values of NDI, ODI, FA, MD, RD, and AD were calculated for each white and gray matter region for each subject. Maps of NDI, ODI, FA, and MD are shown in Fig 1 from two representative participants. Bilateral regions were averaged to provide one measure for each structure.

### Calculation of age-related trajectories

Linear models were used to fit each output parameter with respect to age, controlling for sex, using lm from the R statistical software package. Although brain development is nonlinear over larger age ranges [3, 4], a linear approximation is appropriate for this narrow window from 8 to 13 years. Significance was set at p<0.05, with false discovery rate correction for 14 multiple comparisons corresponding to the 14 brain regions. Slope of the best fit line was calculated. Percent change for each area was calculated as the total change from 8–13 years divided by the mean value.

### Relationships among parameters

To understand relationships between NODDI and DTI parameters, Pearson correlations were performed between each pair of NDI, ODI, FA, MD, and RD across subjects within each white matter tract or subcortical gray matter region of interest, while controlling for sex and age.

### Clustering

Each subject’s NDI and ODI maps were registered to their T1 scan using ANTs (as above), then registered to custom template in MNI space created from 30 healthy children [24]. The rate of change of NDI with age (slope) and the mean NDI and ODI across subjects were calculated for each voxel in MNI space. The first cluster analysis used NDI slope/NDI mean and the second cluster analysis used mean NDI and mean ODI together. A white matter mask from the MNI template, and subcortical regions from the MNI structural atlas were used to limit analysis to white and subcortical gray matter. The Hartigan and Wong algorithm in R [25] was used to group voxels with similar development profiles. The optimal cluster number was determined using the “elbow” of the curve, where increasing the number of clusters provides diminishing reductions to the sum squared error (SSE). Because this determination is subjective, a range of cluster numbers were explored to confirm results.

## Results

### Sex differences

No region showed significant differences between males and females (p<0.05, FDR corrected) on any diffusion metric (NDI, ODI, FA, MD).

### Correlations with age

**Fig 2:**
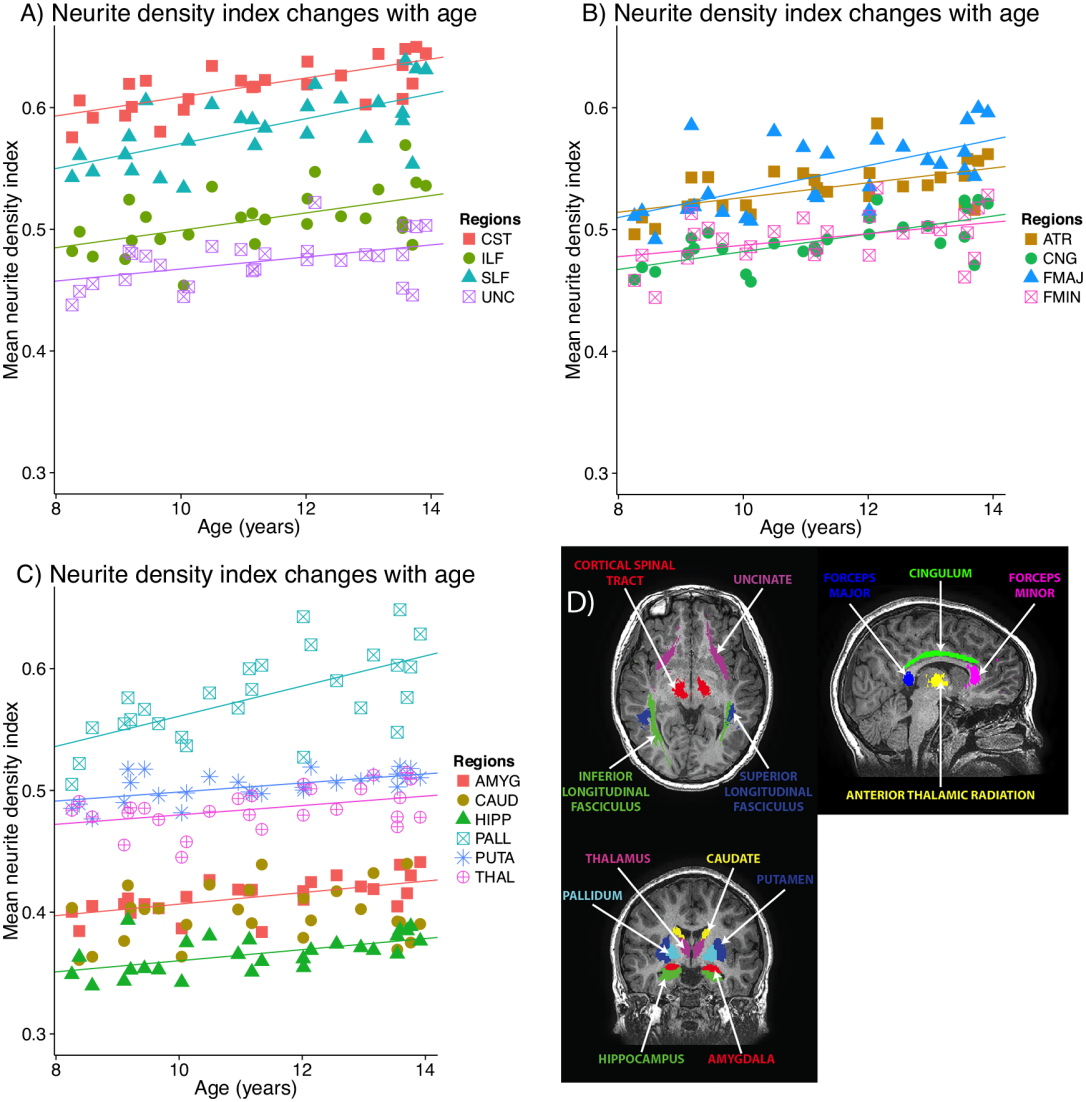
Neurite density index trajectories in white (A, B) and grey (C) matter volumes-of-interest. All regions except the caudate had significant positive correlations with age; significant lines of fit (p<0.05, FDR-corrected) are plotted on the graphs; the best fit line (non-significant) for the caudate is shown as a dotted line. Panel D shows the locations of each region- or tract-of-interest. CST: corticospinal tract; ILF/SLF: inferior/superior longitudinal fasciculus; UNC: uncinate fasciculus; ATR: anterior thalamic radiation; CNG: cingulum; FMAJ: forceps major; FMIN: forceps minor; AMYG: amygdala; CAUD: caudate; HIPP: hippocampus; PALL: pallidum; PUTA: putamen; THAL: thalamus.

All white and grey matter regions except the caudate showed positive correlations between NDI and age (Table 1, Fig 2). The largest percent changes were observed in the pallidum, forceps major, and the SLF (10–13% change, all p<0.001). The smallest percent changes were observed in the forceps minor (5%, p = 0.031), uncinate fasciculus (6%, p = 0.021), and the remaining gray matter regions (putamen, hippocampus, amygdala, thalamus; 3–7% changes, p<0.05). The caudate was the only region with where NDI was not significantly correlated with age.

**Table 1:** Linear fitting results of NDI (neurite density) and ODI (orientation density) vs. age.

NDI							ODI				
Region	% change	Slope (10^−3^/y)	95% CI	Intercept	Sex	R	Slope (10^−3^/y)	95% CI	Intercept	Sex	R
Caudate	3.1	2.07	[-2.7, 6.8]	0.38	-0.012	0.34	-2.91	[-7.2, 1.4]	0.50	-0.017	0.46
Putamen	4.3	3.67**	[1.6, 5.8]	0.46	-0.003	0.61	0.254	[-2.3, 2.8]	0.47	-0.005	0.22
Thalamus	4.7	3.85*	[0.4, 7.3]	0.44	-0.001	0.43	-1.74	[-4.3, 0.9]	0.37	-0.002	0.28
Forceps minor	5.7	4.79*	[0.7, 8.9]	0.44	0.007	0.46	2.94	[-2.4, 8.3]	0.22	0.005	0.24
Uncinate	6.3	5.02*	[1.1, 9.0]	0.42	0.005	0.48	-0.452	[-4.3, 3.4]	0.33	-0.015	0.4
Amygdala	6.7	4.68***	[2.1, 7.3]	0.36	-0.004	0.62	3.59	[-2.1, 9.3]	0.41	-0.010	0.32
ATR	6.8	6.11**	[2.4, 9.8]	0.46	0.005	0.58	-2.73	[-6.8, 1.4]	0.33	-0.004	0.28
Hippocampus	7.2	4.48**	[1.8, 7.1]	0.32	-0.006	0.61	-2.57	[-6.9, 1.8]	0.45	-0.007	0.29
CST	7.5	7.86***	[4.4, 11.3]	0.53	0.005	0.7	-0.334	[-2.2, 1.6]	0.30	-0.010	0.5
ILF	8.2	7.07**	[2.4, 11.7]	0.43	-0.006	0.55	-3.10	[-6.6, 0.4]	0.32	-0.010	0.42
Cingulum	8.9	7.40***	[4.2, 10.6]	0.40	0.008	0.71	-2.80	[-6.8, 1.2]	0.35	-0.014	0.44
SLF	10.3	10.2***	[5.3, 15.1]	0.47	-0.001	0.66	0.138	[-2.4, 2.7]	0.33	-0.011	0.44
Forceps major	11.6	10.7***	[5.4, 16]	0.42	0.004	0.65	1.26	[-3.0, 5.5]	0.25	-0.008	0.25
Pallidum	12.7	12.4***	[6.2, 18.6]	0.44	0.003	0.64	2.13	[-4.7, 8.9]	0.39	-0.021	0.35

Fitting parameters and percent changes for NDI and ODI vs age for each region are shown. Regions are listed in order of increasing percent changes for NDI. All regions except the caudate showed significant age-related changes in NDI; no regions had significant age-related changes in ODI. NDI: neurite density index; ODI: orientation dispersion index; CI: confidence interval; ATR: anterior thalamic radiation; ILF/SLF: inferior/superior longitudinal fasciculus; CST: corticospinal tracts

*: p<0.05,

**: p<0.01,

***: p<0.001.

p-values are corrected for multiple comparisons using false discovery rate.

**Fig 3:**
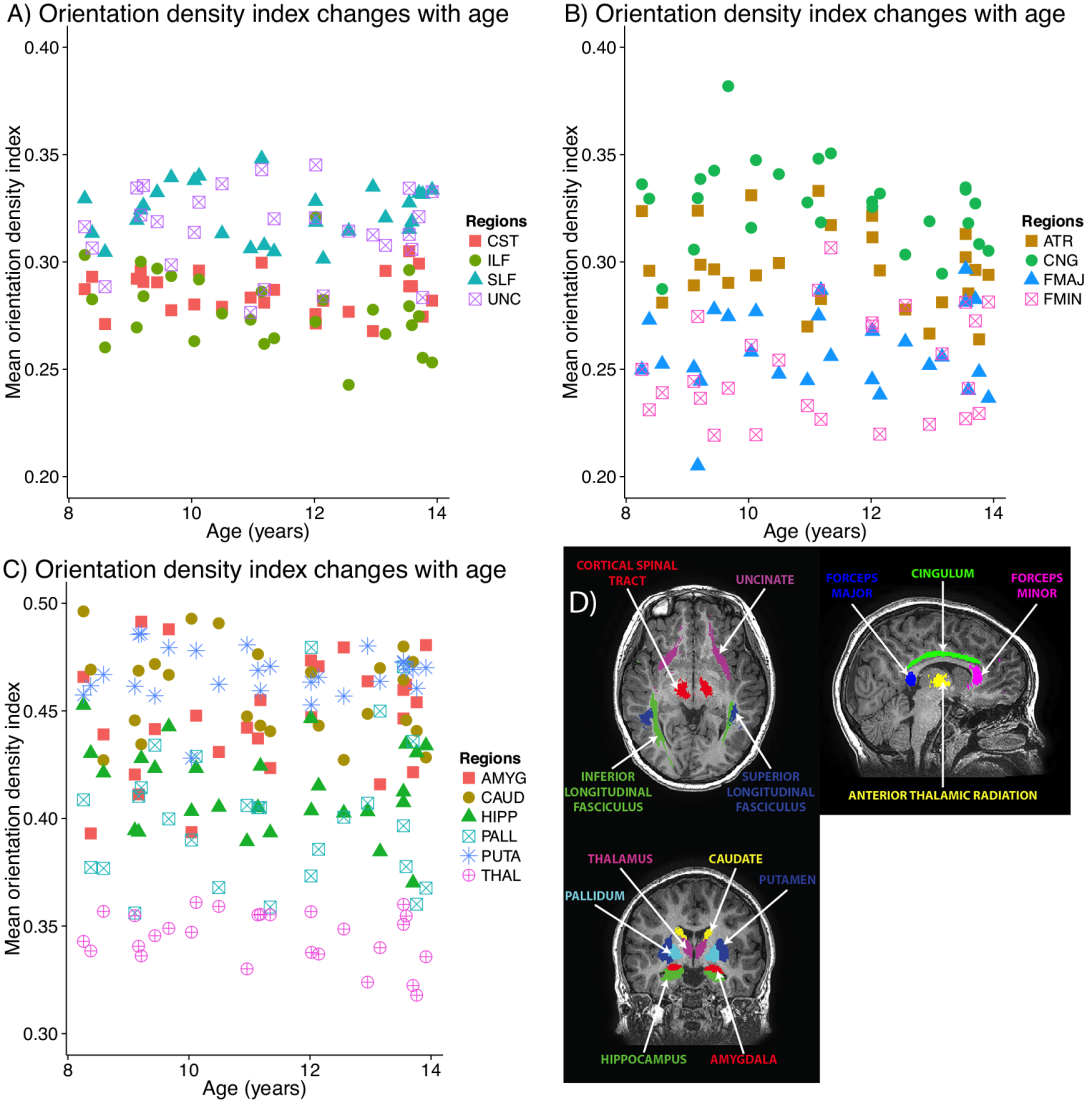
Orientation density index (ODI) trajectories in white (A, B) and grey (C) matter volumes-of-interest. No correlations between ODI and age were significant; non-significant best-fits are shown as dotted lines. CST: corticospinal tract; ILF/SLF: inferior/superior longitudinal fasciculus; UNC: uncinate fasciculus; ATR: anterior thalamic radiation; CNG: cingulum; FMAJ: forceps major; FMIN: forceps minor; AMYG: amygdala; CAUD: caudate; HIPP: hippocampus; PALL: pallidum; PUTA: putamen; THAL: thalamus.

No regions had significant correlations between ODI and age (Fig 3). The cingulum, SLF, and uncinate had the highest values of ODI, while the CST, forceps major, forceps minor, and ILF had the lowest values of ODI. The thalamus showed the lowest values of ODI among grey matter regions.

**Fig 4:**
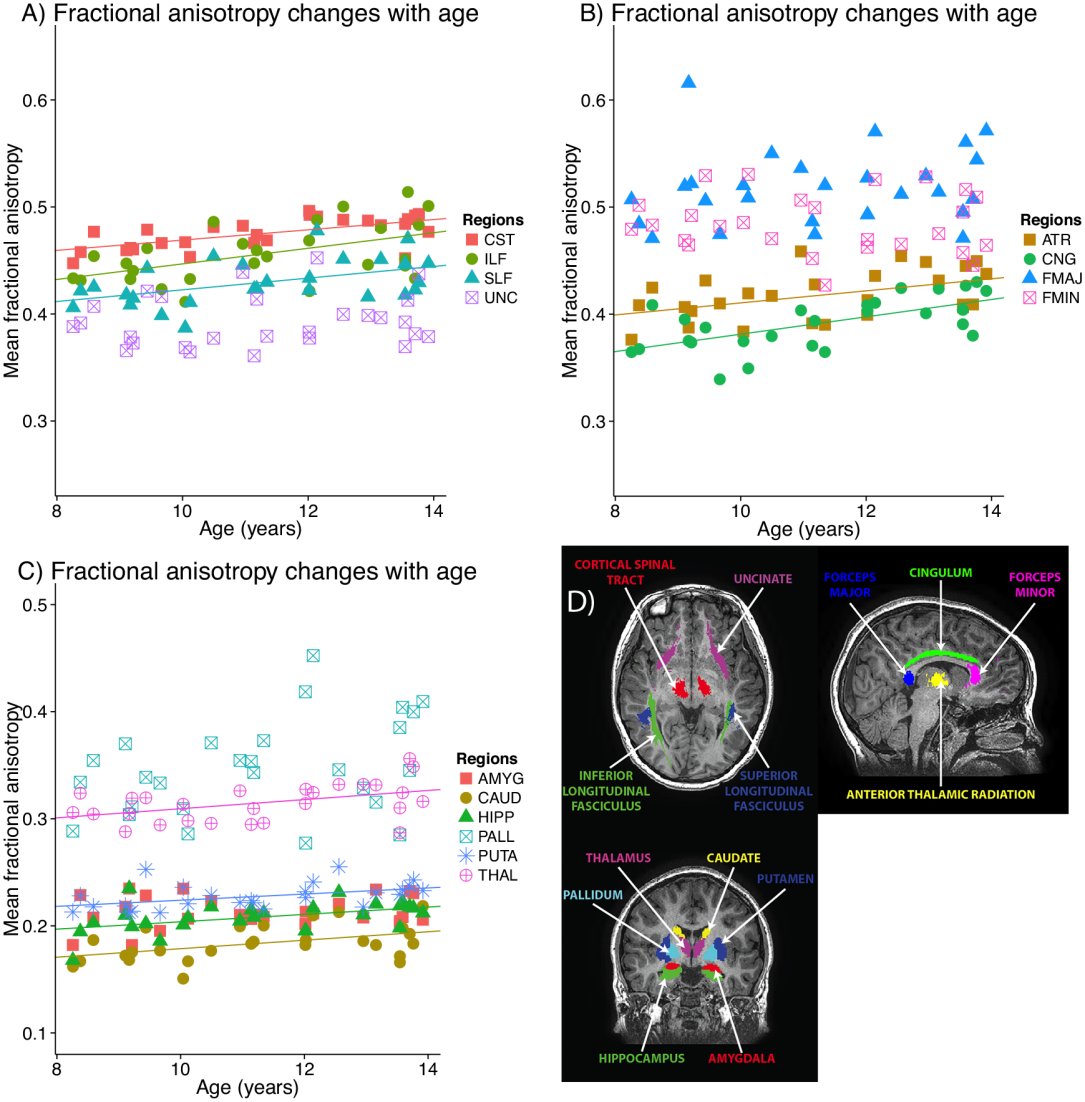
Fractional anisotropy trajectories in white (A, B) and grey (C) matter volumes-of-interest. Most regions had significant positive correlations between age and FA; significant lines of fit (p<0.05, FDR-corrected) are plotted as solid lines. Non-significant best fits are shown as dotted lines. CST: corticospinal tract; ILF/SLF: inferior/superior longitudinal fasciculus; UNC: uncinate fasciculus; ATR: anterior thalamic radiation; CNG: cingulum; FMAJ: forceps major; FMIN: forceps minor; AMYG: amygdala; CAUD: caudate; HIPP: hippocampus; PALL: pallidum; PUTA: putamen; THAL: thalamus.

Most white matter and grey matter regions showed positive correlations between FA and age (Fig 4, Table 2), and negative correlations between MD and RD and age (Table 2). Similar timing profiles were observed with FA and NDI in that most gray matter regions had shallow slopes, while the cingulum and pallidum has the fastest development and the steepest slopes. Notable exceptions are the forceps major and minor, which had high and medium rates of NDI changes, respectively, but no significant correlations between FA and age. All regions except the caudate and thalamus had significant negative correlations between MD and age, with similar timing profiles to the FA changes. Most regions with significant positive FA-age correlations also had significant negative RD-age correlations; there were no regions with significant correlations between AD and age.

**Table 2:** Linear fitting results of FA (fractional anisotropy), mean diffusivity (MD), radial diffusivity (RD), and axial diffusivity (AD) vs. age. Pearson correlation (R) is shown.

	FA	MD	RD	AD
Region	R	R	R	R
Forceps minor	0.08	0.50*	0.26	0.41
Amygdala	0.13	0.60**	0.60**	0.53
Uncinate	0.15	0.48**	0.40	0.34
Forceps major	0.16	0.55**	0.31	0.45
Pallidum	0.37	0.61**	0.54**	<0.01
Caudate	0.46*	0.082	0.13	0.01
Thalamus	0.46*	0.17	0.28	0.11
Hippocampus	0.46*	0.45*	0.51*	0.32
ATR	0.47*	0.50*	0.58**	0.07
Putamen	0.47*	0.50*	0.58**	0.16
SLF	0.49*	0.57**	0.56**	0.48
ILF	0.51*	0.50*	0.56**	0.15
Cingulum	0.63**	0.54**	0.66**	0.01
CST	0.63**	0.56**	0.66**	0.18

The p-values were corrected for multiple comparisons using the false discovery rate method. Regions are listed in order of increasing slope for FA. FA: fractional anisotropy; MD/RD/AD: mean/radial/axial diffusivity; ATR: anterior thalamic radiation; ILF/SLF: inferior/superior longitudinal fasciculus; CST: corticospinal tracts

*: p<0.05,

**: p<0.01,

***: p<0.001

### Correlations among parameters

Partial correlations between FA, MD, RD, NDI, and ODI, controlling for age and sex are reported in Table 3. FA and ODI were significantly negatively correlated in all regions (mean R = -0.74). FA was significantly correlated with NDI in half the regions (mean R = 0.47). In white matter, FA and NDI were positively correlated in the forceps major, inferior and superior longitudinal fasciculus, and the uncinate fasciculus. In gray matter, FA correlated with NDI in the hippocampus, pallidum, and thalamus. MD and NDI were significantly negatively correlated in all regions (mean R = -0.83), while MD correlated significantly with ODI only in the forceps major. RD, a measure typically more closely associated with myelin than FA, was negatively correlated with NDI in all regions (mean R = -0.80), and positively correlated with ODI in half of the regions (mean R = 0.35): the anterior thalamic radiation, forceps major and minor, superior longitudinal and uncinate fasciculi, pallidum, and the thalamus.

**Table 3:** Correlations between NODDI and DTI parameters, controlling for age and sex.

	FA-NDI	FA-ODI	MD-NDI	MD-ODI	RD-NDI	RD-ODI
Region	R	R	R	R	R	R
ATR	0.33	-0.88**	-0.93**	-0.08	-0.80**	0.49*
Cingulum	0.33	-0.78**	-0.74**	-0.22	-0.71**	0.22
CST	0.42	-0.54*	-0.84**	-0.14	-0.80**	0.17
Forceps major	0.78**	-0.90**	-0.91**	0.62*	-0.87**	0.82**
Forceps minor	0.20	-0.92**	-0.77**	-0.15	-0.58*	0.63*
ILF	0.61*	-0.55*	-0.93**	-0.15	-0.85**	0.17
SLF	0.74**	-0.77**	-0.93**	0.31	-0.92**	0.51*
Uncinate	0.51*	-0.87**	-0.90**	0.1	-0.84**	0.55*
Amygdala	0.04	-0.78**	-0.75**	-0.28	-0.76**	-0.07
Caudate	0.31	-0.70**	-0.88**	0.05	-0.89**	0.14
Hippocampus	0.62*	-0.53*	-0.80**	0.08	-0.88**	0.04
Pallidum	0.78**	-0.81**	-0.85**	0.36	-0.87**	0.71**
Putamen	0.23	-0.59*	-0.66**	-0.42	-0.64*	-0.06
Thalamus	0.71**	-0.80**	-0.69**	0.34	-0.89**	0.53*

The p-values were corrected for multiple comparisons using the false discovery rate method. FA: fractional anisotropy; NDI: neurite density index; MD: mean diffusivity; RD: radial diffusivity; ODI: orientation dispersion index; ATR: anterior thalamic radiation; ILF/SLF: inferior/superior longitudinal fasciculus; CST: corticospinal tracts

*: p<0.05,

**: p<0.001

### K-means clustering

The sum of squared error (SSE) vs. the number of clusters was used to determine the optimal number of clusters in the k-means clustering analysis. The “elbow” of the curve, where increasing the number of clusters did not result in a substantial change of SSE, occurred at k = 3. Because choosing the number of clusters is subjective, we also explored the solutions for smaller (k = 2) and larger (k = 4) numbers of clusters.

**Fig 5:**
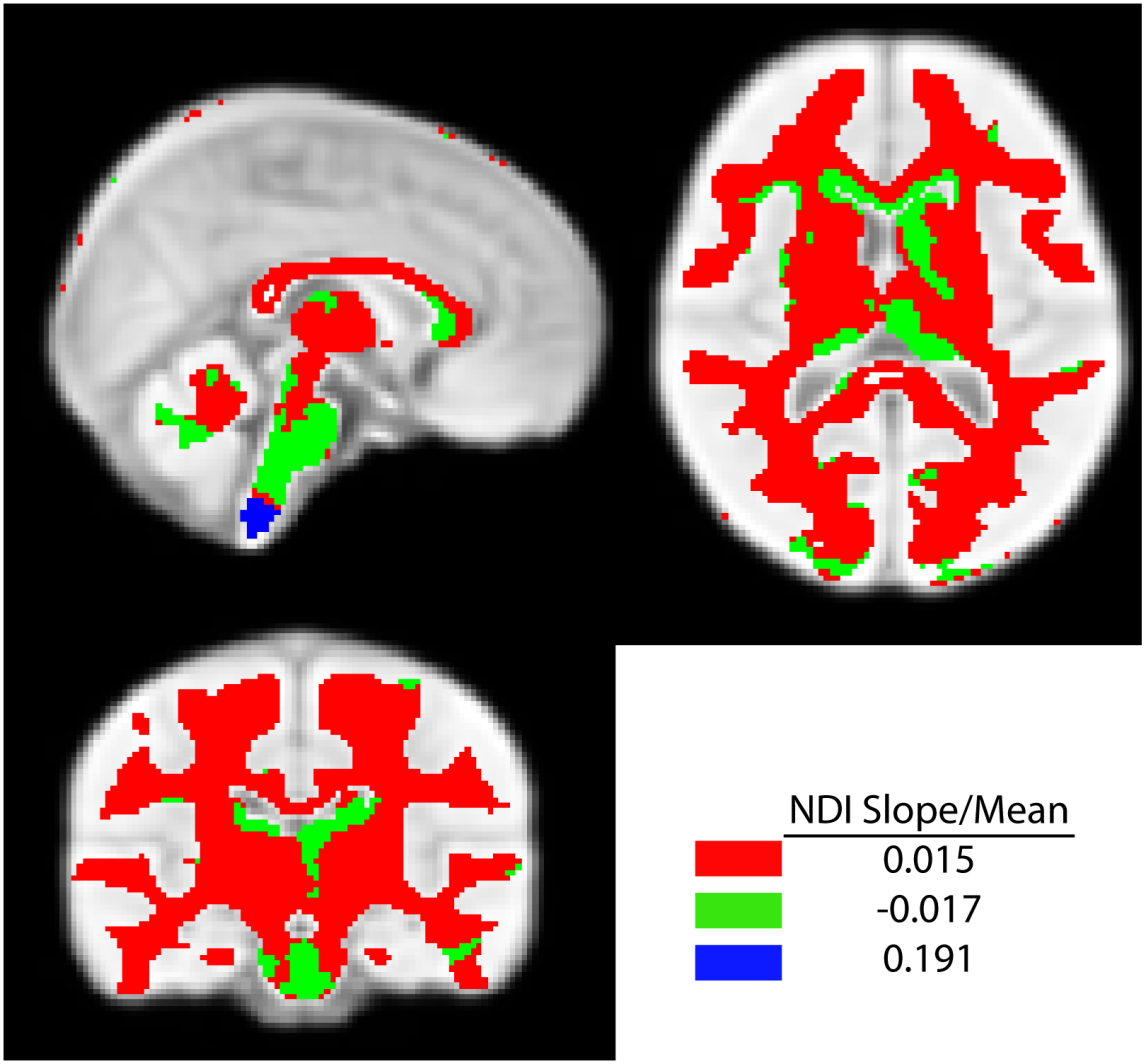
Clusters defined by k-means clustering of NDI slope/NDI mean. Clusters produced with k = 3 are shown on an average brain template. Three clusters were produced, showing positive changes of NDI across most of the brain (red cluster), with small sections of the corpus callosum, corticospinal tract, thalamus, caudate and brainstem havng decreasing NDI with age (green cluster). A third small cluster contained only part of the brainstem and showed large increases of NDI.

Clusters generated using k = 3 are shown in Fig 5 for NDI slope/NDI mean, with the average values for each cluster. Cluster 1 (red in Fig 5) was a large cluster containing most of the white and subcortical gray matter. Cluster 2 (green) contained the posterior part of the genu of the corpus callosum, and sections of the thalamus, caudate, and brainstem. This cluster had a negative NDI slope/NDI mean, indicating decreases with age. Cluster 3 contained only a few voxels in the lower brainstem with a very large value of NDI slope/NDI mean. Results using alternate numbers of clusters were similar (S1 Fig). The 2-cluster solution combined clusters 1 and 2, forming one large cluster containing almost all white and subcortical gray matter, and another cluster containing a small section of the lower brainstem. The 4-cluster solution provided similar groups to the 3-cluster solution, but further separated much of the subcortical gray matter and some subcortical white matter from other brain areas.

**Fig 6:**
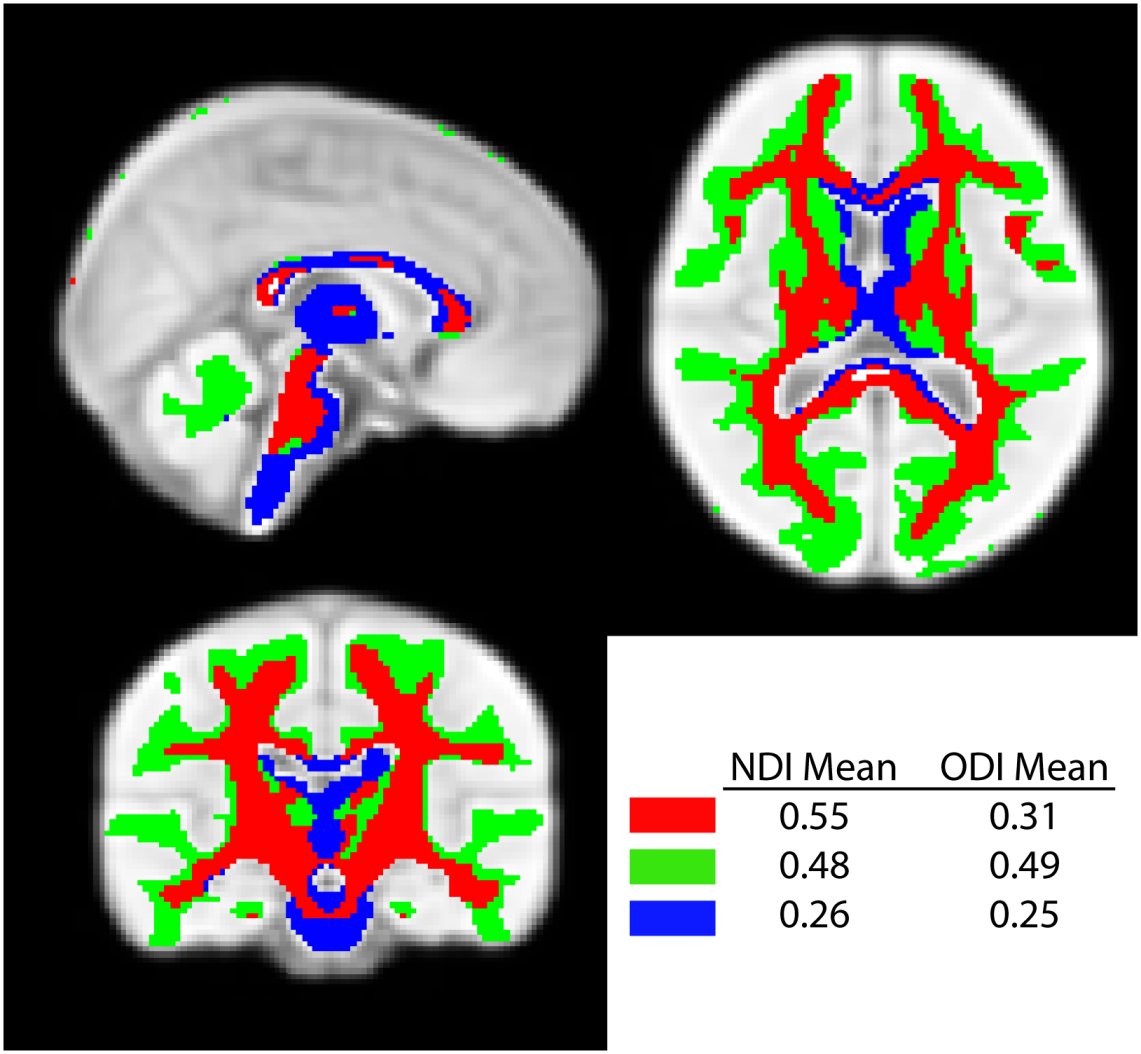
Clusters defined by k-means clustering of NDI and ODI means. Clusters produced with k = 3 are shown on an average brain template. Clusters separated the subcortical (green) from central white matter (red), with a third cluster with low NDI and low ODI values containing parts of the corpus callosum, thalamus, caudate, and brainstem.

Fig 6 shows clusters generated using k = 3 for the NDI and ODI means together. The first cluster (red in Fig 6) contained the central white matter and part of the thalamus and brain stem, and showed high values of NDI with medium values of ODI. A second cluster (green) contained the subcortical white matter, some subcortical gray matter, and the cerebellum; this cluster had high values of both NDI (though not as high as cluster 1) and ODI. The final cluster (blue) contained a large part of the thalamus, part of the corpus callosum, and much of the brainstem, and had the lowest values of both NDI and ODI. The solution using 2 clusters (S2 Fig) separated only subcortical and central white matter, with both clusters containing subcortical gray matter structures. The 4-cluster solution separated an additional layer of white matter, parsing white matter tracts into subcortical, intermediate, and central regions.

## Discussion

Using DTI and NODDI, we demonstrate age-related changes across the brain. The age-related increases of FA and decreases of MD observed agree well with the growing literature on diffusion changes during childhood and adolescence, and our NODDI results suggest that these are primarily driven by increasing myelination and/or axonal packing, rather than changes in axon coherence and geometry. FA and NDI are sensitive to similar underlying physiological changes, such as myelination and axonal packing, and similar trends were found in both parameters versus age. However, stronger correlations (larger R^2^ values) with age were found for NDI than for any of the DTI parameters (FA, MD, RD, AD), suggesting that NDI is more sensitive to age-related changes during late childhood and early adolescence. This confirms findings of a recent study showing that NDI is a better marker of maturation in childhood and adolescence than FA [12]. The multi-component model used to estimate NODDI parameters makes it unsurprising that NDI is more closely associated with microstructural changes than DTI parameters, as the contributions of other factors (e.g., axon coherence) that do not change with age are eliminated from NDI but represented in DTI parameters. Interestingly, however, when correlations between metrics were examined, ODI was more closely correlated with FA across participants, suggesting a strong link between the two parameters in terms of variation across individuals, even if ODI does not change with age. Previous studies in infants describe similar findings of positive correlations between NDI and FA, and negative correlations between ODI and FA [15], with stronger correlations between FA and ODI than FA and NDI [26]. In our study, MD was closely linked with NDI but not significantly correlated with ODI for most regions. Because MD is a measure of total water movement, it makes sense that it is related to neurite density, with higher density restricting diffusion more, rather than related to the orientation and geometry of tracts. RD, generally thought to be more closely associated with myelin than FA [9, 27], was strongly correlated with NDI in all regions, and correlated with ODI in half of the regions examined. This suggests that RD is strongly related to neurite density across participants (including the level of myelination), but that axon coherence also plays a role, particularly in the white matter and more ordered subcortical gray matter structures (pallidum and thalamus).

Our NODDI results show significant regional variation in development trends. The steepest slopes and largest percent changes, representing the fastest development across this age range, were observed in the SLF, forceps major, and pallidum. Other subcortical gray matter structures and the uncinate fasciculus had the shallowest slopes. These white matter findings are consistent with previous DTI studies showing slower but more prolonged development in the uncinate fasciculus, but faster development in callosal areas, and intermediate rates in the CST and SLF [1, 3, 4, 28]. The two previous NODDI studies do not report slopes for all regions, but appear to observe the same general pattern, with steeper slopes in the SLF than the uncinate [12], or in callosal areas than association tracts [11]. In our study, no areas had significant correlations between ODI and age, suggesting that axon coherence in all regions remains stable across this age range.

Most studies only examine white matter, though one NODDI study demonstrated increasing NDI in the thalamus in infants [15]. We observed relatively slower development of most subcortical gray matter areas compared to white matter, though the pallidum had a large change of NDI across the age range. A previous DTI study showed significant increases of FA and/or decreases of MD in the caudate, putamen, and globus pallidus in late childhood; the thalamus did not show significant diffusion changes in childhood, but children had higher MD than adults, suggesting continued development [14]. Our NODDI results suggest that changes within subcortical gray matter, as in white matter, are due to increasing neurite density rather than changes in geometry.

The clustering results provide further insight into microstructural patterns across the brain. The clustering of NDI slope/NDI mean revealed that much of the white matter is developing similarly, with modestly increasing NDI values across our age range. However, parts of the posterior genu of the corpus callosum, CST, caudate, thalamus, and brainstem formed a cluster with a negative NDI slope, suggesting decreases in axonal density. It is important to note that averaging across each of the CST and thalamus produced positive correlations of NDI with age, and averaging across the caudate showed no correlations with age, suggesting that development varies across these structures. Previous studies overwhelmingly find increasing FA and decreasing MD with age in children [29], though some studies do show slight decreases of FA or increases of MD in childhood or adolescence in limited areas [1, 4]. In agreement with our findings, a previous study showed decreasing magnetization transfer ratios, another measure sensitive to myelin, in the CST of adolescent males [30]. Negative correlations between NDI and age may be due to increasing axon size or increasing extra-cellular space, both of which would cause the neurite density to appear to decrease. Partial volume averaging with voxels containing high CSF could also drive down NDI values, and development of extra-cellular space could cause a decline in intra-cellular space. The final cluster contained only a small section of the lower brainstem, which had very high increases of NDI; however, this area can be prone to image artifacts and registration errors, so future studies will need to verify this result. Overall, the clustering results suggest similar patterns of development throughout most of the brain, with localized areas of NDI decreases. A previous NODDI study reported different maturation patterns in core white matter, subcortical, and cortical areas from childhood to older adulthood [11]. Our results indicate a slightly more homogenous pattern of development, but that should be expected over a much narrower age range. In contrast to some previous DTI studies [31, 32], we did not observe any obvious posterior-to-anterior or inferior-to-superior pattern of maturation.

Using the mean values of NDI and ODI, a hierarchical pattern was clearly identified, with core white matter having higher NDI and lower ODI values than subcortical white matter, reflecting the fact that central white matter tracts are the most myelinated, tightly packed, and organized sections. Sections of the corpus callosum, thalamus, and caudate formed a third cluster with very lower NDI and ODI values. Interestingly, this cluster overlapped with the cluster from Fig 5 that showed a negative NDI slope, suggesting different patterns of both microstructure and its development over time in these areas.

This study has several limitations. Importantly, white matter development during this time period is not linear, though the narrow age range in our study allowed a linear approximation of the development trends. Furthermore, the cross-sectional nature of this study precludes the measurement of change over time, instead limiting us to inferences based on correlations between imaging parameters and age. Finally, data-driven clustering is limited by our small sample size. Future studies with wider age ranges, longitudinal data points, and/or more subjects may be able to better characterize patterns of diffusion parameter maturation across the brain, identify any subtle changes of ODI, and characterize the contributions of factors including sex and cognitive abilities.

In conclusion, this work helps further characterize healthy white and grey matter development during late childhood and early adolescence using NODDI, an advanced diffusion imaging and modeling technique. Data-driven clustering revealed increasing NDI values with age across the brain, with only small parts of structures showing decreasing NDI. Overall, using NODDI allowed for a more specific characterization of white matter and subcortical gray matter development than using DTI parameters alone, suggesting that the FA increases observed in many previous studies are driven by increases of myelination and/or axonal packing, but not axon coherence.

## Supporting information

S1 Fig Clusters produced with k = 2 and k = 4 for NDI slope/NDI mean. Clusters produced with k = 2 (A) and 4 (B) for NDI slope/NDI mean are shown on an average brain template. The 2-cluster solution combined clusters 1 and 2, forming one large cluster containing almost all white and subcortical gray matter, and another cluster containing a small section of the lower brainstem. The 4-cluster solution provided similar groups to the 3-cluster solution, but further separated much of the subcortical gray matter and some subcortical white matter from other brain areas.
(TIF)

S2 Fig Clusters produced with k = 2 and k = 4 for NDI and ODI means. Clusters produced with k = 2 (A) and 4 (B) for NDI and ODI means are shown on an average brain template. The 2-cluster solution separated only subcortical and central white matter, with both clusters containing subcortical gray matter structures. The 4-cluster solution separated an additional layer of white matter, parsing white matter tracts into subcortical, intermediate, and central regions, with subcortical gray matter and the brain stem making up most of the other cluster. All solutions suggest an inner-to-outer profile of white matter microstructural properties.
(TIF)
