# Supplementary figures and images for: Detailing neuroanatomical development in late childhood and early adolescence using NODDI
Source: PLoS One. 2017 Aug 17;12(8):e0182340. doi: 10.1371/journal.pone.0182340 (PMC5560526; doi:10.1371/journal.pone.0182340)

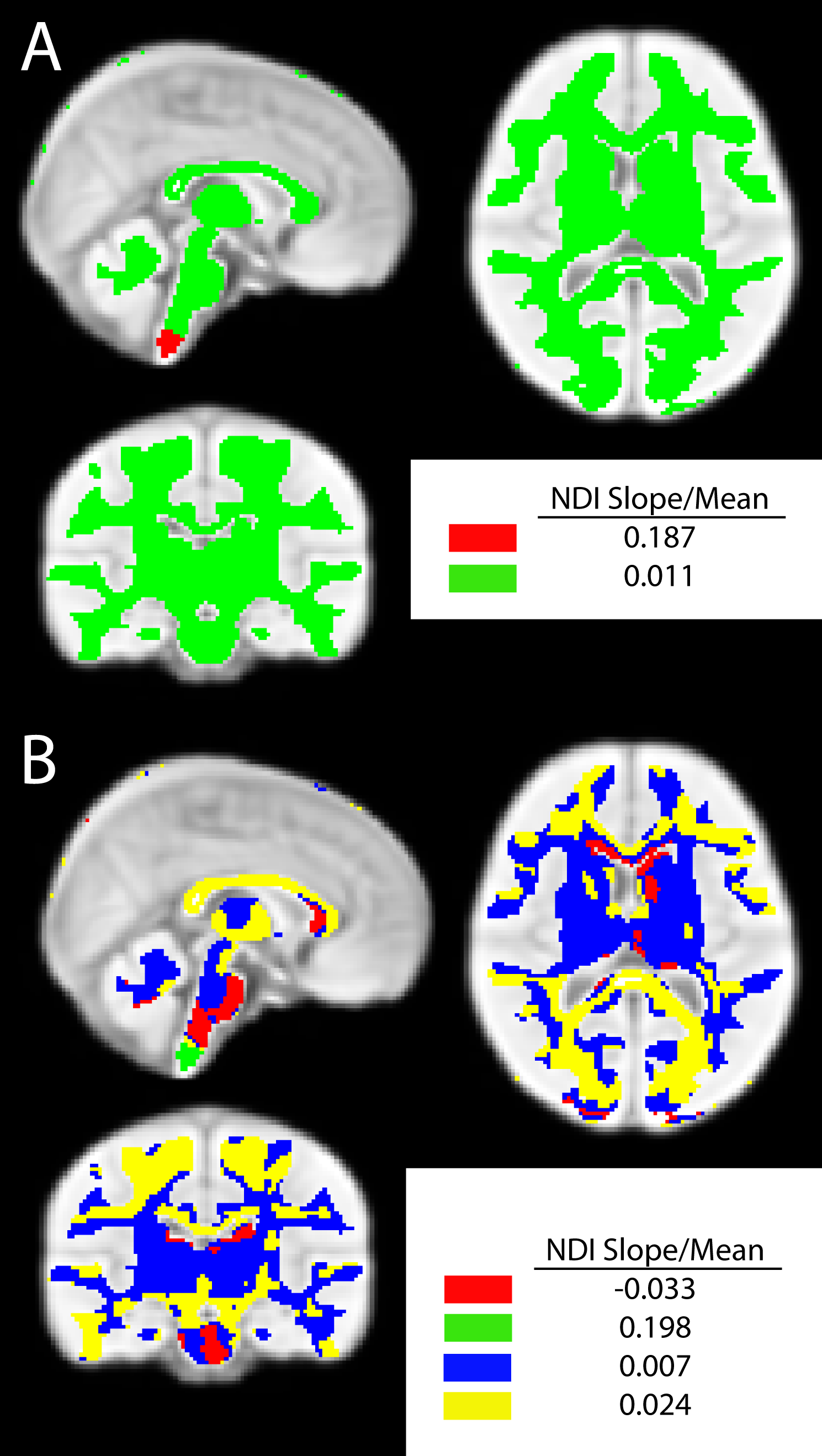

Supplement: S1 Fig — Clusters produced with k = 2 (A) and 4 (B) for NDI slope/NDI mean are shown on an average brain template. The 2-cluster solution combined clusters 1 and 2, forming one large cluster containing almost all white and subcortical gray matter, and another cluster containing a small section of the lower brainstem. The 4-cluster solution provided similar groups to the 3-cluster solution, but further separated much of the subcortical gray matter and some subcortical white matter from other brain areas. (TIF) [file pone.0182340.s001.tif]

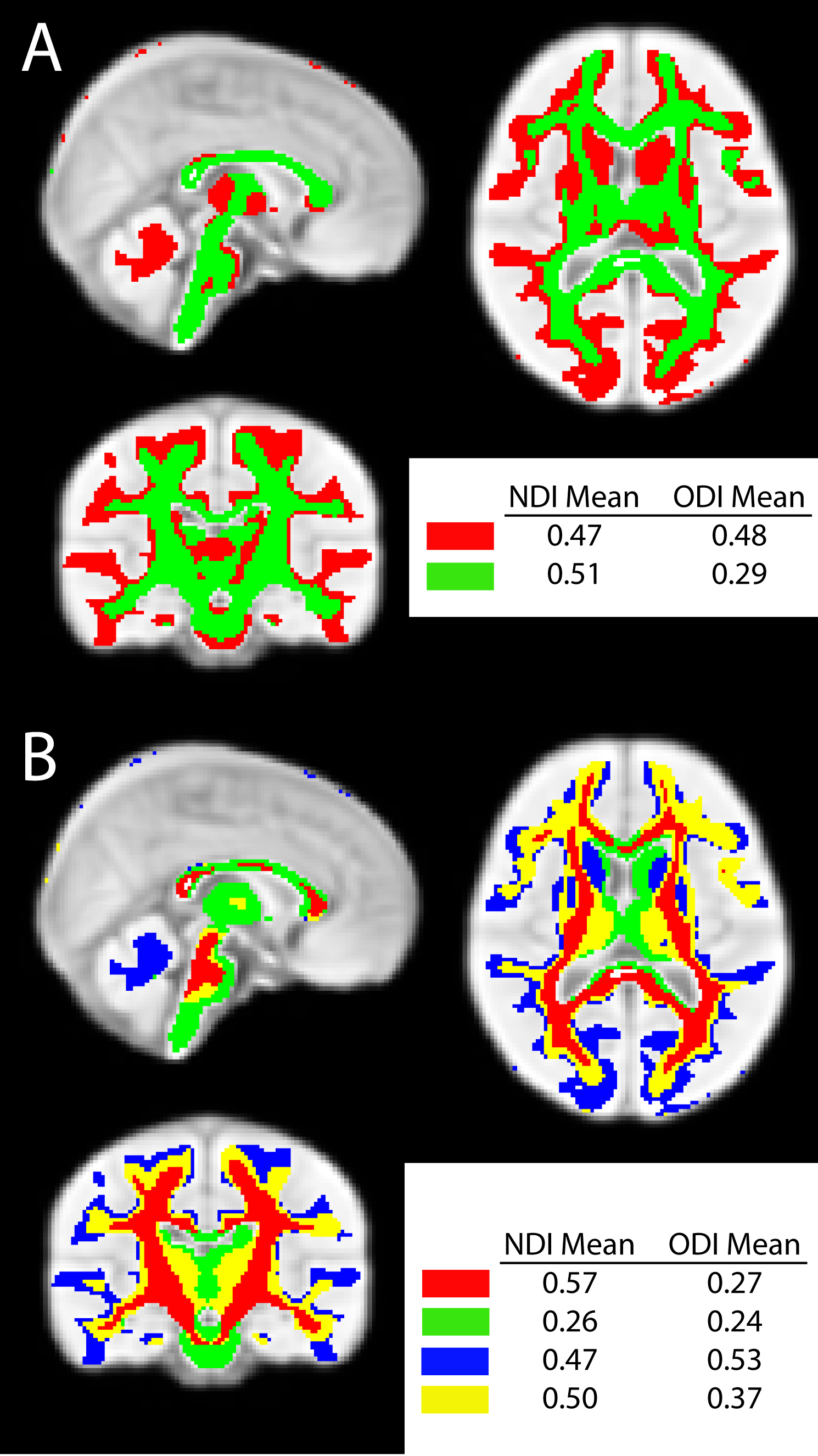

Supplement: S2 Fig — Clusters produced with k = 2 (A) and 4 (B) for NDI and ODI means are shown on an average brain template. The 2-cluster solution separated only subcortical and central white matter, with both clusters containing subcortical gray matter structures. The 4-cluster solution separated an additional layer of white matter, parsing white matter tracts into subcortical, intermediate, and central regions, with subcortical gray matter and the brain stem making up most of the other cluster. All solutions suggest an inner-to-outer profile of white matter microstructural properties. (TIF) [file pone.0182340.s002.tif]
